# Post-inflammatory behavioural despair in male mice is associated with reduced cortical glutamate-glutamine ratios, and circulating lipid and energy metabolites

**DOI:** 10.1038/s41598-020-74008-w

**Published:** 2020-10-08

**Authors:** Shi Yu Chan, Fay Probert, Daniel E. Radford-Smith, Jennifer C. Hebert, Timothy D. W. Claridge, Daniel C. Anthony, Philip W. J. Burnet

**Affiliations:** 1 Department of Psychiatry, Warneford Hospital, University of Oxford, Warneford Lane, Oxford, OX3 7JX UK; 2 Department of Pharmacology, University of Oxford, Oxford, OX1 3QT UK; 3 Department of Chemistry, University of Oxford, Oxford, OX1 3TA UK; 4 Present Address: Psychosis Neurobiology Lab, McLean Hospital, Belmont, MA 02478 USA

**Keywords:** Biomarkers, Biological techniques, Biological models, Metabolomics, Neuroscience, Emotion, Neuroimmunology

## Abstract

Post-inflammatory behaviours in rodents are widely used to model human depression and to test the efficacy of novel anti-depressants. Mice injected with lipopolysaccharide (LPS) display a depressive-like phenotype twenty-four hours after endotoxin administration. Despite the widespread use of this model, the mechanisms that underlie the persistent behavioural changes after the transient peripheral inflammatory response remain elusive. The study of the metabolome, the collection of all the small molecule metabolites in a sample, combined with multivariate statistical techniques provides a way of studying biochemical pathways influenced by an LPS challenge. Adult male CD-1 mice received an intraperitoneal injection of either LPS (0.83 mg/kg) or saline, and were assessed for depressive-like behaviour 24 h later. In a separate mouse cohort, pro-inflammatory cytokine gene expression and 1H nuclear magnetic resonance (NMR) metabolomics measurements were made in brain tissue and blood. Statistical analyses included Independent Sample t-tests for gene expression data, and supervised multi-variate analysis using orthogonal partial least squares discriminant analysis for metabolomics. Both plasma and brain metabolites in male mice were altered following a single peripheral LPS challenge that led to depressive-like behaviour in the forced swim test. The plasma metabolites altered by LPS are involved in energy metabolism, including lipoproteins, glucose, creatine, and isoleucine. In the brain, glutamate, serine, and N-acetylaspartate (NAA) were reduced after LPS, whereas glutamine was increased. Serine-modulated glutamatergic signalling and changes in bioenergetics may mediate the behavioural phenotype induced by LPS. In light of other data supporting a central imbalance of glutamate-glutamine cycling in depression, our results suggest that aberrant central glutaminergic signalling may underpin the depressive-like behaviours that result from both inflammation and non-immune pathophysiology. Normalising glutaminergic signalling, rather than seeking to increase serotonergic signalling, might prove to be a more coherent approach to the development of new treatments for mood disorder.

## Introduction

Depression is a common illness affecting those of all ages and disposition. Despite a surge in the availability and prescription of antidepressants over the last three decades, the prevalence of the disorder continues to increase, and the global health burden remains substantial^1^. Psychological stress, a key contributor to depression and other psychiatric conditions^2^, alters the neuroimmune system, particularly through the induction of a pro-inflammatory state with cytokine signalling initiated by activated microglia^3–6^. In humans, inflammatory biomarkers are associated with depression and depressive symptomology^7,8^, and attenuating this immune activation may be a prerequisite for antidepressant treatment efficacy^9^.

A systemic injection of lipopolysaccharide (LPS) generates a peripheral and central pro-inflammatory response, mimicking the outcomes of stress-induced neuroinflammation. In humans, inducing inflammation with LPS resulted in increased anxiety and depressed mood, and deficits in memory tasks^10^. In rodents, intraperitoneal administration of LPS also results in anxiety- and depressive-like behaviour^11–14^. These behavioural deficits are reversed with antidepressants^15^.

Inflammation-related behavioural deficits are paralleled by increases in plasma pro-inflammatory cytokines, which peak 2 to 4 h after LPS administration and normalize to control levels 24 h post-LPS. However, brain concentrations of pro-inflammatory cytokines remained elevated 24 h after the LPS administration^16^. While it is clear from previous investigations and our current work that the peripheral administration of LPS results in depressive-like behavioural changes, the underlying mechanisms remain poorly understood. Several mechanisms may underlie how locally expressed cytokines in the central nervous system (CNS) alter neuronal function. The induction of indoleamine 2,3-dioxygenase (IDO) by pro-inflammatory cytokines interferon gamma and tumour necrosis factor α (TNFα) increases kynurenine levels and its downstream metabolites^11,17^. Quinolinic acid production in activated microglia may then contribute to the excessive excitability of N-methyl-D-aspartate (NMDA) receptors and altered neuronal plasticity in inflammation-associated depression^18,19^. Direct effects of cytokines on glutamate metabolism in the CNS are also mediated in part by astrocyte activation^20^, whereby net glutamate release is increased, further exacerbating NMDAR excitotoxicity^21^. Increased cortical levels of TNFα and interleukin (IL)-1β may also reduce the levels of brain derived neurotrophic factor (BDNF)^22,23^. BDNF critically regulates glutamate release and neurotransmission through its receptors^24^, and levels are reduced in individuals with depression^25^. It is also pertinent to note that microglia and basally expressed cytokines directly contribute to normal synaptic plasticity via neuronal receptors, and their alteration during neuroinflammation can directly promote maladaptive neuroplasticity and depression^4,26,27^. The study of the brain metabolome, in combination with multivariate statistical analysis, affords an opportunity to explore key biochemical changes that may also contribute to pathophysiology in inflammation-associated depressive-like behaviour.

The metabolome is a collection of small molecule metabolites, that include amino acids, lipids, carbohydrates and nucleic acids, the relative levels of which can reflect the influence of both genetic and environmental factors, such as diet and stress^28,29^. Global metabolite profiling of a sample, also referred to as untargeted metabolomics, serves as an unbiased method of measuring detectable metabolites. This approach has proven useful in identifying metabolic profiles that are characteristic of a given phenotype. For instance, a metabolic signature of social ranking and susceptibility to depressive-like behaviour was proposed in a mouse chronic social defeat stress model^28^. Metabolomics has also been applied to explore inter-individual variation in survival outcomes after a high-dose LPS administration in rats. In this instance, susceptibility to LPS was mainly associated with lipid metabolism, where different species of lysophosphatidylcholines were found in the survival and non-survival groups, and sphingosine was unique to the survival group. Although the study focused on inflammation-induced fatality rather than behavioural changes, the results suggested that LPS administration affects components of energy metabolism, such as S-adenosyl methionine (SAM), and key Krebs cycle constituents glucose and citrate^30^.

The peripheral and central metabolomic effects of low-grade, sub-febrile inflammation have not been explored, but are important to investigate because some psychiatric illnesses have been associated with increased levels of pro-inflammatory cytokines in both serum and cerebrospinal fluid (CSF), without accompanying markers of infection^31^. Therefore, the aims of the current study were to 1) confirm that peripherally administered LPS has central effects on brain inflammatory markers and increases depressive-like behaviour after 24 h in mice, and 2) to determine what are the plasma and brain metabolite profiles that accompany the behavioural changes. An examination of the biochemical pathways perturbed as a result of the inflammatory stimulus at this dose will provide further insight into how inflammation might translate to behavioural changes and identify potential new targets to ameliorate the pathophysiology of neuroinflammation-induced dysregulation of emotion.

## Methods

**Figure 1:**
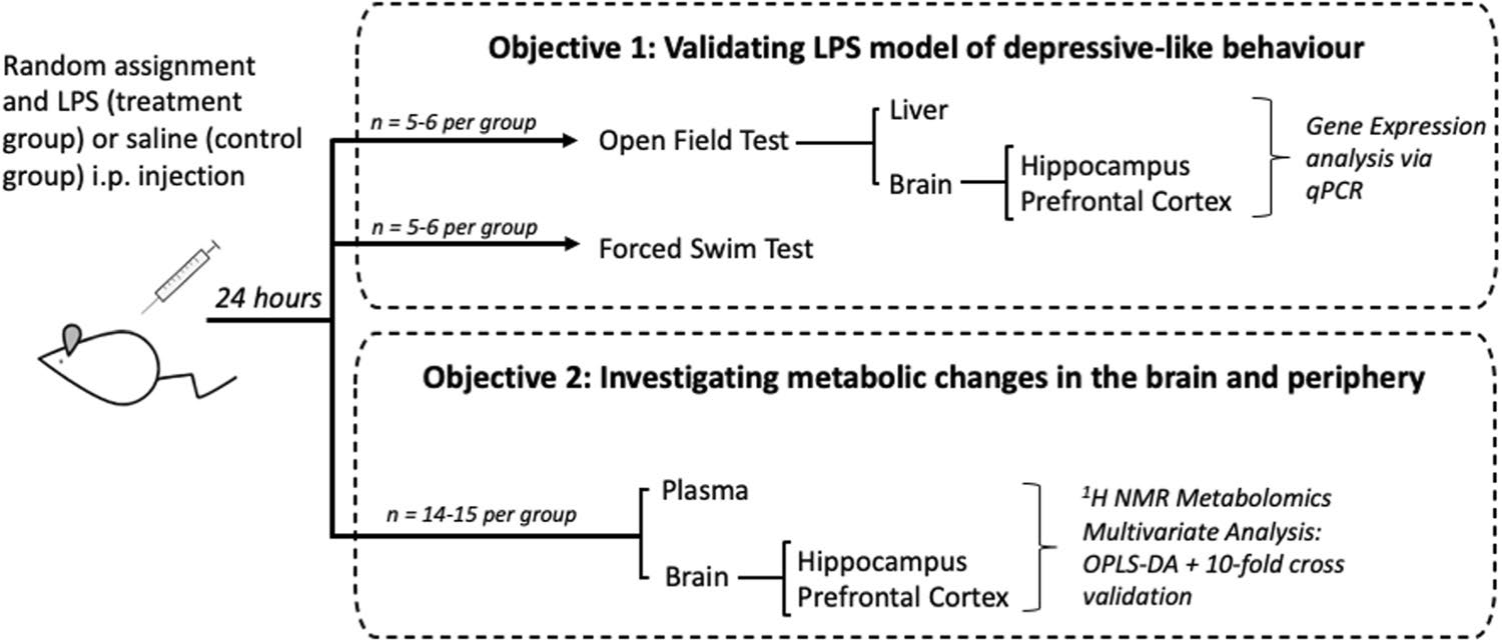
Summary of study design. Mice were treated with lipopolysaccharide (LPS) or saline to fulfil the study objectives of (A) validating the LPS model of depressive-like behaviour with behavioural and mRNA analysis and (B) studying peripheral and central metabolic changes with ^1^H NMR metabolomics. Original artwork was provided by Dr Biyan Zhang, A*STAR, Singapore.

The study design is summarised in Fig. 1.

### Animals and treatments

All animal procedures were carried out in accordance with UK Home Office Animals (Scientific Procedures) Act (1986) and associated Home Office guidelines. The local Animal Welfare and Ethical Review Body at the University of Oxford approved the procedures specific to this study. The experiments were performed on adult, male CD-1 mice (30-35 g, Charles River). All mice were cohoused from juvenile age, four per cage, under standard environmental conditions (21 ± 1 °C, 12-h light–dark cycle, lights on 0700, humidity 50 ± 5%), and all had access to food and water ad libitum. Mice were randomly and equally allocated to treatment (LPS) and control (saline) groups.

A single intraperitoneal (i.p.) injection of LPS (0.83 mg/kg) from *Escherichia coli* serotype O111:B4, (Sigma Aldrich), was used to stimulate an inflammatory response. This method has previously been used to induce depressive-like behaviours in mice at this timepoint^32,33^. Mice in the control group received an equivalent volume of sterile saline via the same route of administration. The numbers of mice to be used for each experiment (Fig. 1) were chosen according to the biological variance observed in our previous studies that had employed i.p LPS (at the same dose and timepoint)^32–36^. Male mice were used to match the methodology used in our previous work^37^ and that of others^33,38^, and to avoid the confounding effects of sex on metabolomics^39^.

### Behaviour

Depressive-like behaviour was evaluated in the forced swim test (FST) paradigm 24 h after intraperitoneal administration of LPS or saline. Behaviour in the open field test (OFT) was assessed in a separate mouse cohort immediately prior to tissue collection (Fig. 1), approximately 23 h after LPS injection. Mice were placed in the room used for behavioural testing and given time to habituate to the new environment for 30 min prior to the start of testing. All behaviour was videotaped using a 1080p web camera (Huafu HI-Tech, China), and analysed post-hoc by a trained observer blinded to treatment groups. Animals were tested in a controlled, randomised fashion with regard to treatment groups, with minimal noise disruption.

#### Open field test

Mice were individually placed into a corner of a dimly lit (10 lx) black open arena (84 × 57 x 40cm, *l x w x h*) and were allowed to explore the enclosure freely for 5 min. ANY-maze mouse-tracking software (Stoelting Co., Illinois, U.S.) was used to automatically measure the distance travelled, time spent in centre, and immobility time. The number of rearing events, including both unsupported and supported against the walls of the enclosure, were counted manually as an indication of exploratory behaviour^40–42^.

#### Forced swim test

A modified FST was employed as previously described^43^. Mice were gently placed in a transparent container (32.5 × 14.5 x 13cm, *l x w x h*) filled to a depth of 9 cm with 30 ± 1 °C warm water, from which there were no means of escape. Room lighting was set to 25 lx. Immobility, or floating behaviour, was defined as the absence of movement for longer than 2 s other than that required to stay afloat. Total time immobile and the latency to first become immobile were scored manually. Each mouse was in the apparatus for a total of five minutes. The first two minutes were not scored to avoid the effect of stress-induced hyper-locomotion^43^.

### Tissue collection

All animals were culled 24 h after LPS administration between 12-1 pm. Dietary intake is minimal at this time because CD1 mice spend much of this period asleep^44^. As a consequence, mice rarely display spikes in their blood glucose or insulin levels. We ensured that this was consistent across all mice used in the study. Tissue was not collected from mice that underwent the FST. Brain prefrontal cortex and hippocampus were immediately harvested, snap-frozen in isopentane on dry-ice, and stored at − 80 °C for downstream gene expression and metabolomic analyses. Previous studies have shown that LPS induces robust changes in the mRNA and protein levels of pro-inflammatory cytokines and/or NMDA receptors in the prefrontal cortex and hippocampus^16,37,45–47^. In addition, trunk blood was collected in Ethylene Diamine Tetra Acetic Acid (EDTA) tubes and centrifuged for 15 min at 5000 rpm. Plasma was isolated and stored at − 80 °C prior to metabolomics analysis. Liver was also collected for gene expression analyses to assess levels of peripheral inflammatory markers.

### RNA extraction and reverse transcription

Brain and liver tissue samples (50-100 mg) were homogenized in 1 ml TRI-reagent (Sigma), and total RNA was extracted following manufacturer’s instructions, and dissolved in nuclease-free water (NF H_2_O). For reverse transcription, RNA was diluted to 250 ng/ul in NF H_2_O, and treated with RNase-free DNase (Promega), for 20 min at 37 °C, followed by 5 min at 75 °C. A reverse transcription (RT) reaction mix (1 μL of 10X RT buffer, 0.4 μL 25 mM dNTP mix, 1 μL 10X random primers, 0.5 μL RNase inhibitor, 1 μL reverse transcriptase, 1.1 μL NF H2O) was added to 5 μL of each sample for a final concentration of 100 ng/μL. This mixture was then incubated for 1 h at 37 °C.

### Quantitative polymerase chain reaction (qPCR)

qPCR was performed using a QuantStudio 6 Flex Real-Time PCR system (Thermo Fisher Scientific) in 384-well format. Each sample was run in triplicate as a 12 μL reaction consisting of 1 μL forward primer, 1 μL reverse primer, 6 μL SYBR Green (Thermo Fisher Scientific), and 4 μL of sample (12 ng template cDNA). Relative gene expression of serum amyloid A2 (SAA2), IL-10 and TNFα were determined in the liver. The abundance of IL-1β, IL-6, and TNFα mRNAs were investigated in the brain. Beta-2-microglobulin^48^ (B2M) was used as the reference gene for liver samples, and transferrin receptor^49^ (TFRC) was used as the reference gene for brain samples. The mRNA level of these genes was not affected by i.p. LPS administration (data not shown). Primer sequences are summarized in Table S1.

### Metabolomics

#### Perchloric acid extraction

The protocol for brain tissue metabolite extraction was adapted from a previously described method^50^. Briefly, brain tissue metabolites were extracted in a solution of 0.1 N hydrochloric acid HCl/Methanol (200% v/w), 0.02 N HCl (1000% v/w) and 3 M perchloric acid (250% v/w) with a glass mortar and pestle. Samples were then centrifuged at 3500 ×*g* for 15 min at 4 °C, and supernatants containing aqueous-phase metabolites were transferred to a new tube.

Supernatants were then neutralized in 10 M potassium hydroxide KOH (92.2% v/w) and cold 0.5 M phosphate buffer (276% v/w) and adjusted to pH = 7. The neutralized solution was then left on ice for 15 min and centrifuged for an additional 15 min at 3500 x*g* at 4 °C. Supernatants were then transferred to a fresh tube and lyophilized in a Savant Speedvac concentrator (ThermoFisher Scientific, UK). Lyophilized samples were stored at − 80 °C until further analysis.

#### ^1^H Nuclear Magnetic Resonance (NMR) Spectroscopy

Sample preparation was performed as previously described^51–53^. Briefly, plasma samples were defrosted on ice and centrifuged at 17,000 x*g* for 5 min at 4 °C. An equal volume of plasma was aliquoted (100 μL) into a fresh tube and diluted to 600 μL in a 75 mM phosphate buffer (5:1 disodium phosphate Na_2_HPO_4_, monosodium phosphate NaH_2_PO_4_ in 100% D_2_O, pH 7.4). The volume of plasma used was limited by the volume of plasma collected, and the volume chosen was the maximum volume available for 90% of samples.

Lyophilized brain tissue samples were resuspended in 600 μL of phosphate buffer (0.2 M Na_2_HPO_4_, 0.043 NaH_2_PO_4_, in 100% D_2_O with 0.05 wt % 3-trimethylsilylpropanoic acid (TSP)).

^1^H NMR spectra were acquired using a 700-MHz Bruker AVII spectrometer operating at 16.4 T equipped with a ^1^H (13C/15 N) TCI cryoprobe^52^. Sample temperature was stable at 310 K. ^1^H NMR spectra were acquired using a one-dimensional (1D) Nuclear Overhauser Effect Spectroscopy (NOESY) pre-saturation scheme for attenuation of the water resonance with a 2 s pre-saturation^52^. An additional sequence, the spin-echo Carr-Purcell-Meiboom-Gill (CPMG) sequence, was used for plasma samples to suppress broad signals arising from large molecular weight plasma components with a τ interval of 400 μs, 80 loops, 32 data collections, an acquisition time of 1.5 s, a relaxation delay of 2 s, and a fixed receiver gain^51^. CPMG spectra provide a measurement of small molecular weight metabolites and mobile side chains of lipoproteins in the plasma sample and were used for all further analysis of plasma samples.

#### NMR data processing

Processing methods were adapted from previously published methods and parameters^51,52^. NMR spectra were imported into MestreNova (Mestrelab Research, Spain) and each spectrum was then processed manually with phase 0 (PH0) correction, baseline correction (Bernstein polynomial fit, order = 3), and referencing to an added standard (TSP referenced to δ0) for brain tissue, and an internal standard (Lactate referenced to δ1.33) for plasma. The individual spectra were then stacked, and binned (sum method, width of each integral region = 0.02 ppm). Binning refers to a function where the whole spectrum is divided into bins of equal width, and all the peaks in each bin is integrated to obtain a value representing the area of all the peaks in a bin.

Inter-individual variation was reduced by total area normalization and TSP normalization (brain tissue only). Noise areas were determined using spectral relative standard deviation (RSD) values^34^ and the addition of the mean and standard deviation for each bin. Broadly, areas that were removed include the water peak, regions before 0.7 ppm, regions after 9.38 ppm, noise region 5.0 to 6.0 ppm, and contamination EDTA peaks for plasma samples. The metabolomics pipeline is summarised in Fig S1, and further details on processing methods can be found in supplementary methods section 2.

### Statistical analysis

#### Behaviour and mRNA expression

An independent sample t-test was used to compare means between the LPS and saline groups. Results were expressed as mean ± standard error of the mean (SEM), with Cohen’s *d* value reported as effect size. Equality of variances was tested with Levene’s Test and when this assumption was not met, significance with equal variances not assumed was reported instead. Square root transformation was used to achieve normality of skewed data, only in the latency to immobility in the FST. Statistical analyses were performed using the psych^54^ and lsr^55^ packages in R.3.3.2 and SPSS v20. 5–6 mice per group were used in the analyses of behaviour and gene expression (Fig. 1).

#### Metabolomics

Preliminary exploratory data analysis: Normalized bin values were imported into SIMCA (Umetrics, Sweden). Data was visualised initially with principal component analysis (PCA) scores plots with pareto scaling^56^.

Model building: Supervised multi-variate analysis was then conducted using orthogonal partial least squares discriminant analysis (OPLS-DA) to produce predictive models and identify metabolites driving the discrimination between treatment groups^57^.

OPLS-DA models were built in R 3.3.2^58^ using the ROPLS package^29^ and an in-house R script using tenfold cross validation repeated for a total of 100 iterations, producing an ensemble of 1000 models. The main outcome of interest is the predictive accuracy of the model, reported as mean predictive accuracy of the 1000 models built with the standard error mean (standard deviation/$\sqrt {100}$ where 100 is the number of iterations). This determines how accurate the model is at predicting which group each sample belongs to, thus assessing the discriminatory power of the model. Other parameters assessed the sensitivity of the models and reported the goodness of fit through average Q^2^ values, R^2^X values, and R^2^Y values.

Model Validation: OPLS-DA models were validated by repeating the cross-validation process with group labels randomly permuted. This ensemble of OPLS-DA models, representing the null distribution, was used to calculate the predictive accuracy achieved by random chance. Further details on processing methods can be found in supplementary methods section 3.

Metabolite identification and direction of change: If a model of LPS vs saline was significantly better at predicting sample groups than a random model with ~ 50% accuracy, the variable importance in projection (VIP) scores were used to identify the key bins that were important for building that model. Bins with high VIP scores were considered to contain discriminatory metabolites.

Metabolites were assigned to peaks in these bins through a combination of literature values^59,60^, reference to the human metabolome database (HMDB)^61^, and confirmation with two-dimensional (2D) correlation spectroscopy (COSY). COSY provides confirmation that peaks occupying different positions on the spectrum belong to the same metabolite through cross-peaks that show a correlation between signals. The direction of change between groups was also determined by comparing the means of metabolite levels between LPS-treated animals and saline controls in SPSSv20 (independent samples T-test). A total of 14–15 mice per group were used in the analyses of metabolomic data (Fig. 1).

## Results

### Validation of LPS-induced depressive-like behaviour

**Figure 2:**
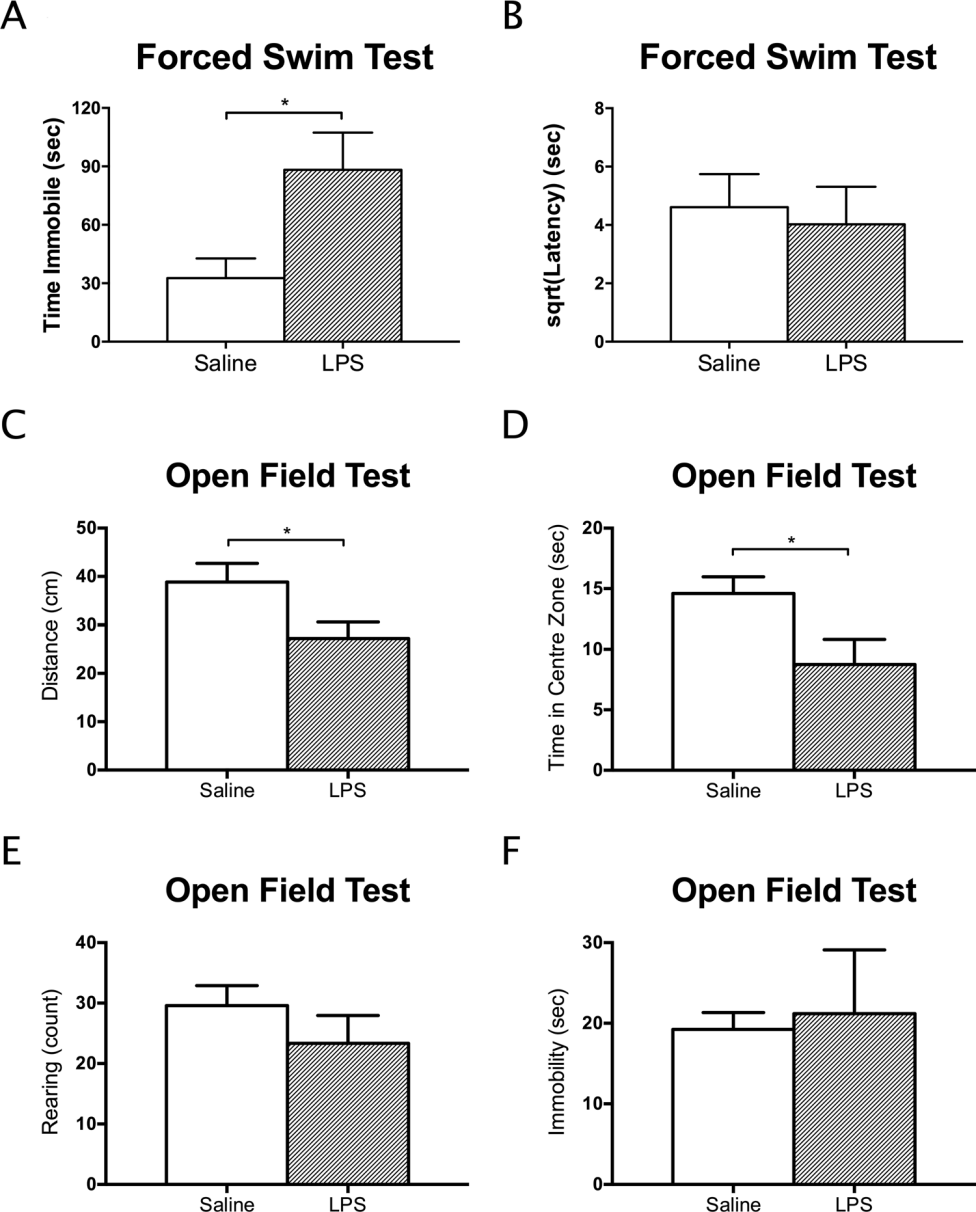
A single injection of LPS increased immobility time in the forced swim test (**A**) relative to mice injected with saline only. Latency to first become immobile was not affected (**B**). In the open field test, both the average distance travelled (**C**) and time in the centre zone (**D**) were reduced, suggesting that the LPS injection was also anxiogenic after 24 h. Rearing (**E**) and immobility time (**F**) in the open field, other measures of locomotor activity, were not altered after 24 h. Data presented as mean + SEM. *p < 0.05; **p < 0.01.

The single administration of LPS significantly increased immobility time in the FST relative to saline (T_1,7.467_ = 2.59, *p* = 0.0344, mean difference = 55.64, CI: 5.38 to 105.90, cohen’s *d* = 1.47, Fig. 2A), a well-documented measure of depressive-like behaviour. Latency to immobility was not affected (*p* > 0.50; Fig. 2B). Locomotor activity in a novel environment was assessed by the mean distance travelled in the OFT. Immobility time and time spent in the centre zone were assessed as indicators of anxiety-like behaviour. The number of rearing events assessed exploratory behaviour which is sensitive to malaise associated with CNS inflammation^41,42^. Rearing and immobility time in the OFT did not differ between the two groups (*p* > 0.30; Fig. 2E and F). However, distance travelled (T_1,9.792_ = 2.27, *p* = 0.0470, mean difference = 11.61, CI: 0.19 to 23.03, cohen’s *d* = 1.31), and time spent in centre zone (T_1,8.341_ = 2.35, *p* = 0.0457, mean difference = 5.850, CI: -0.06 to 11.76, cohen’s *d* = 1.39) was reduced after LPS treatment (Fig. 2C and D).

**Figure 3:**
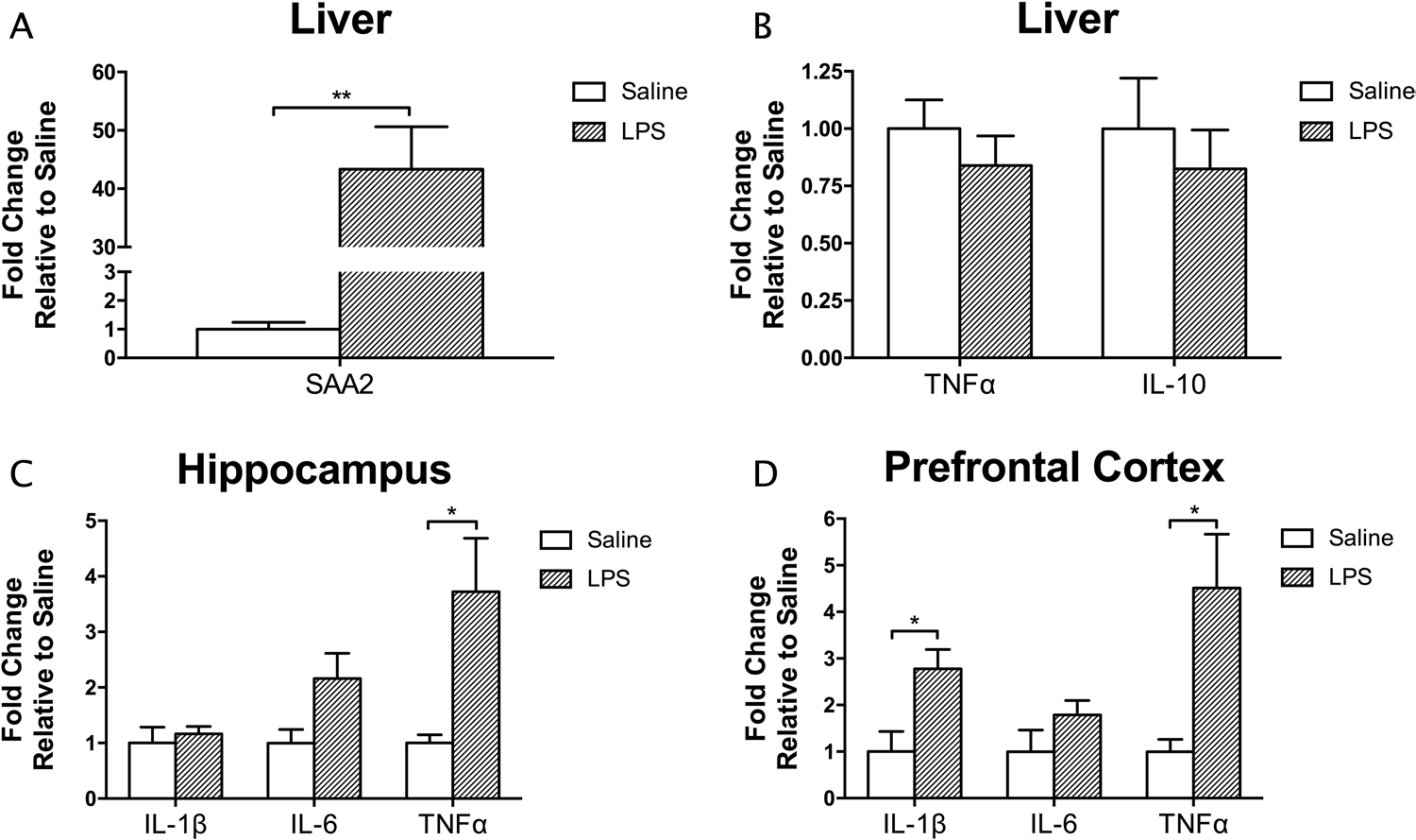
A single injection of LPS increased the expression of liver SAA2 (**A**), but did not alter liver cytokine expression (**B**) after 24 h. On the other hand, increased expression of inflammatory cytokines in the brain at this timepoint was evident after endotoxin administration. This was more pronounced in the prefrontal cortex (**D**), which had significantly increased expression of TNFα and IL1ß in the LPS group, compared to the hippocampus (**C**), where only TNFα expression was elevated. Data presented as mean + SEM. *p < 0.05; **p < 0.01.

The expression of established inflammatory markers was measured in both liver and brain tissue to validate the peripheral and central inflammatory effects, respectively, of a single injection of LPS relative to saline. Post- LPS administration increased hepatic SAA2 mRNA (T_1,5.011_ = 5.836, *p* = 0.002, mean difference = 4234.97%, CI: 2370.98% to 6098.95%), a major acute phase protein (APP) of the acute phase response (APR) (Fig. 3A). However, no differences were found between treatment groups for TNFα or IL-10, indicating that LPS-mediated cytokine changes in the liver had returned to baseline after 24 h (Fig. 3B). In the brain, LPS administration increased the expression of inflammatory cytokines in both the hippocampus and prefrontal cortex (Fig. 3C and D). In the hippocampus, there was a significant increase in TNFα mRNA (T_1,5.239_ = 2.79, *p* = 0.037, mean difference = 272.25%, CI: 24.78% to 519.72%). In the prefrontal cortex, the mRNA of both IL-1β (T_1,7_ = 2.93, *p* = 0.022, mean difference = 177.5%, CI: 34.1% to 320.91%) and TNFα (T_1,8_ = 2.41, *p* = 0.043, mean difference = 351.16%, CI: 14.64% to 687.68%) were increased.

### Plasma metabolomics

**Figure 4:**
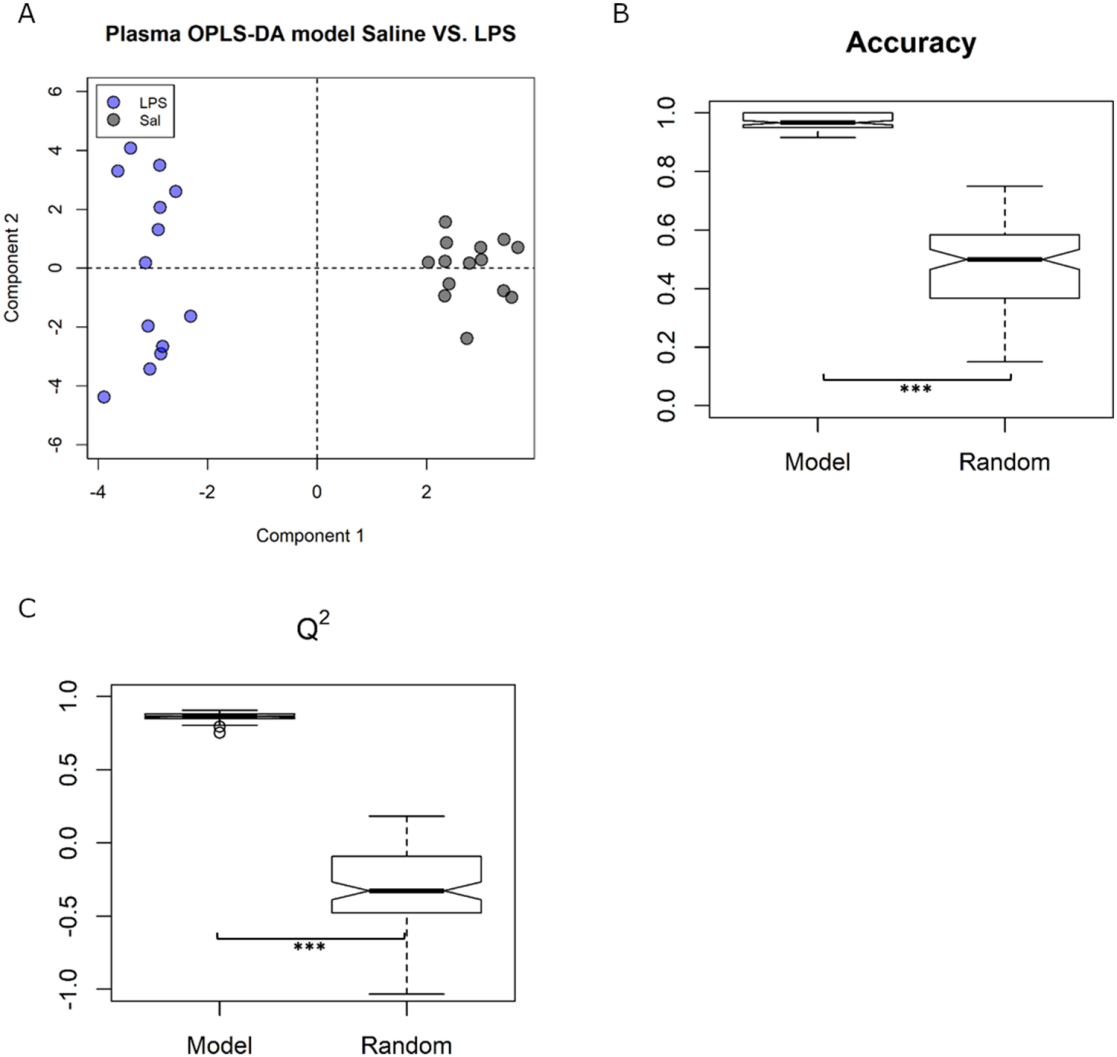
Plasma Saline/LPS models showed (**A**) good discrimination between treatment groups when treatment was set as class (OPLS-DA). (**B**) Predictive accuracy and (**C**) Q^2^ measures were both significantly higher in the ensemble of Saline/LPS models compared to the null models with group labels randomly permuted. ***p < 0.001.

To study the effect of inflammation on plasma metabolites, untargeted OPLS-DA was used to discriminate between animals with and without LPS treatment (Fig. 4A). A tenfold cross validation scheme, followed by 100 iterations, created an ensemble of 1000 models that had a mean predictive accuracy of 97 ± 1.1%. These models were significantly more accurate than null models (with randomly permuted group labels) at predicting which treatment group a plasma sample belonged to (mean predictive accuracy 49%, *p* < 0.001, Fig. 4B). Likewise, in other model validation measures such as the Q^2^ (0.86 ± 0.007 compared to −0.31 ± 0.032, *p* < 0.001, Fig. 4C), sensitivity (+ 41%, *p* < 0.001), and specificity (+ 37%, *p* < 0.001), the Saline/LPS models were significantly better than the randomly permuted null models (Fig S4 and Table S2). This confirms that a single dose of LPS resulted in significant changes to plasma metabolites that persist until 24 h.

**Figure 5:**
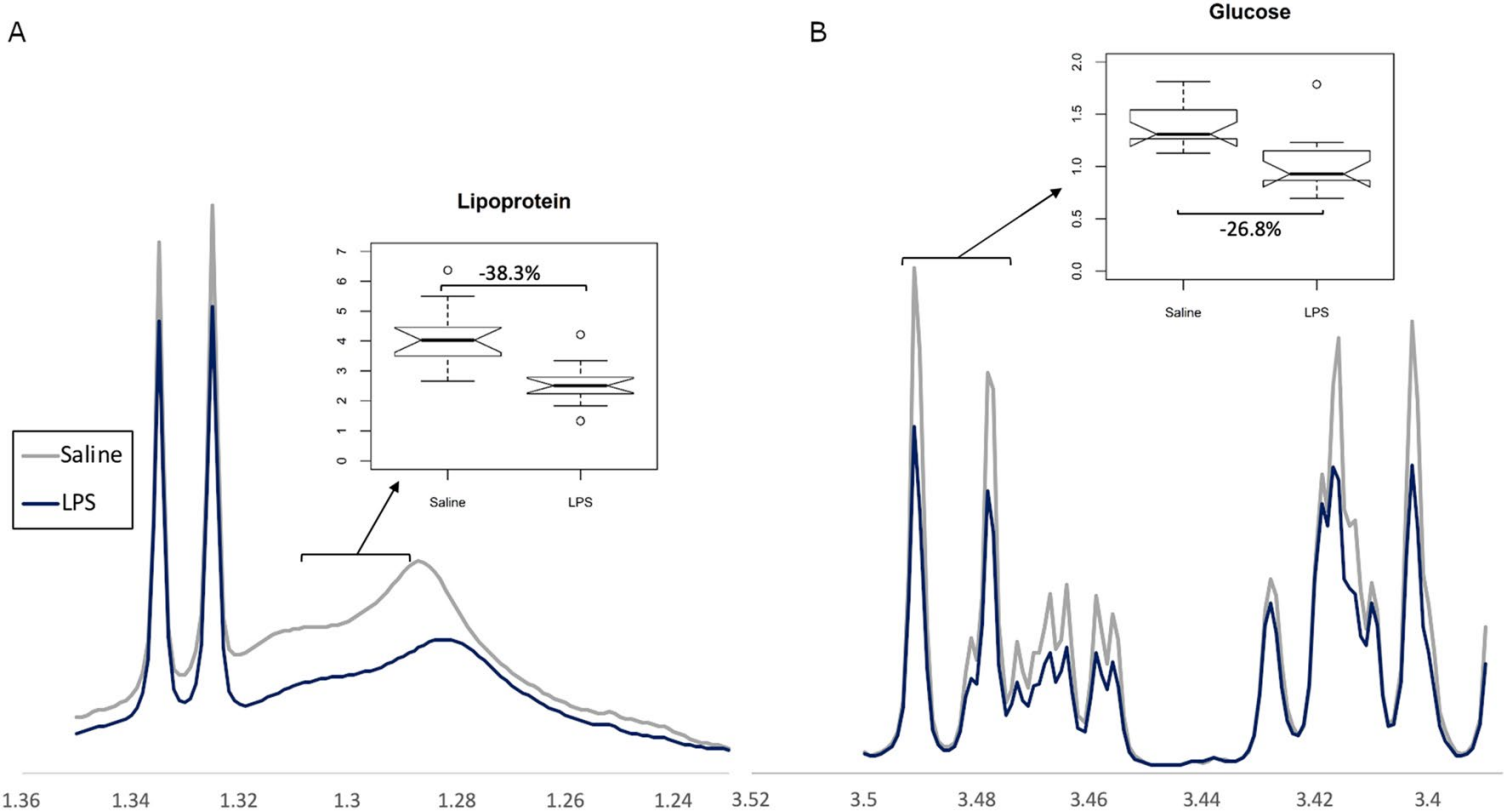
Representative images of NMR spectra showing group differences between (**A**) a lipoprotein bin and (**B**) a glucose bin. Spectra expressed as average of treatment group.

The key components contributing to the models were identified based on their VIP scores and summarized in Table 1. LPS treatment resulted in decreased levels of lipoproteins (Fig. 5A) and glucose (Fig. 5B), and increased levels of creatine and isoleucine. No endotoxin peaks were observed in the spectra, thus changes observed were attributed to downstream effects of inflammation.

**Table 1:** Summary of the key metabolites driving the discrimination between treatment groups, and their direction of change compared to saline.

Key plasma metabolites	Direction of change (LPS vs Saline)
–CH_3_ mobile lipoprotein (HDL)	↓**
–CH_3_ mobile lipoprotein (VLDL)	↓*
Isoleucine	↑***
Isoleucine/Leucine	↑***
(–CH_2_–)_n_ mobile lipoprotein	↓**
α-glucose	↓***
β-glucose	↓***
Creatine	↑*

*HDL* high density lipoprotein, *VLDL* very low density lipoprotein *p < 0.05; **p < 0.01; ***p < 0.001.

### Brain metabolomics

**Figure 6:**
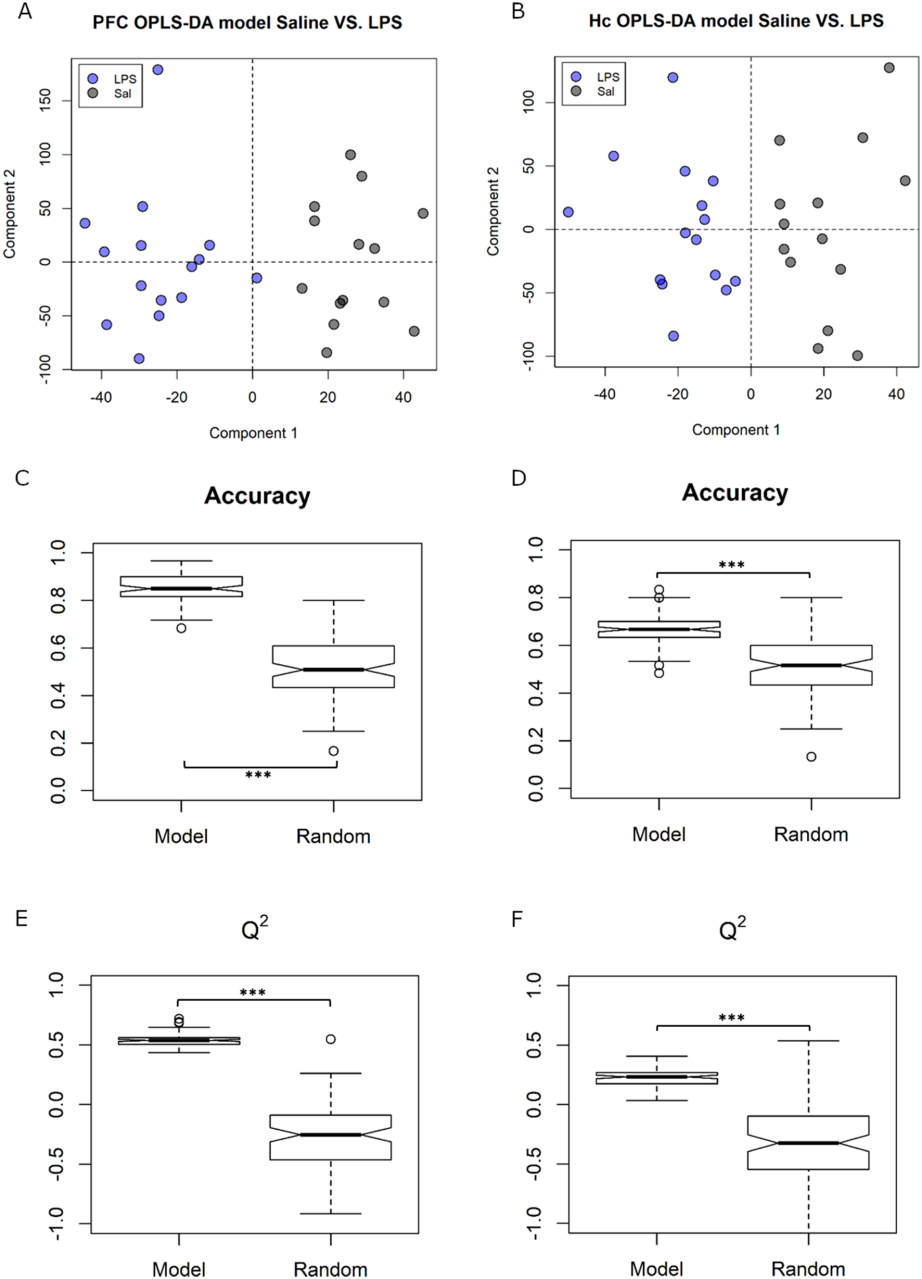
Brain Saline/LPS models showed (**A** and **B**) good discrimination between treatment groups when treatment was set as class (OPLS-DA) in both PFC and Hc. (**C** and **D**) Predictive accuracy and (**E** and **F**) Q^2^ measures were both significantly higher in the ensemble of Saline/LPS models compared to the null models with group labels randomly permuted. ***p < 0.001.

To determine the central effects of peripherally-induced inflammation, untargeted OPLS-DA was next used to discriminate between animals treated with and without LPS in two brain regions – the prefrontal cortex and hippocampus (Fig. 6A and B). Predictive accuracy of the ensemble of 1000 models built was significantly higher than that of null models with randomly permuted group labels in both brain regions. In the prefrontal cortex, LPS/Saline models were able to predict which treatment group a sample belonged with an accuracy of 85.3 ± 2.18%, compared to 51.1 ± 3.12% in null models (*p* < 0.001, Fig. 6C). Likewise, LPS/Saline models in the hippocampus had a predictive accuracy of 67.1 ± 2.8% compared to 50.7 ± 3.06% in null models (*p* < 0.001, Fig. 6D). The LPS/Saline models also performed better than the randomly permuted null models on all other model validation measures such as the Q^2^ (hippocampus: 0.225 ± 0.016 compared to −0.343 ± 0.043; prefrontal cortex: 0.537 ± 0.009 compared to −0.315 ± 0.04, both *p* < 0.001, Fig. 6E and F), sensitivity (hippocampus: + 18.7%, prefrontal cortex: + 33.0%, both *p* < 0.001), and specificity (hippocampus: + 16.9%, prefrontal cortex: + 29.8%, both *p* < 0.001; Fig S5 and S6). Thus, a single dose of LPS, administered in the periphery, altered the composition of aqueous metabolites in brain tissue 24 h after treatment.

**Figure 7:**
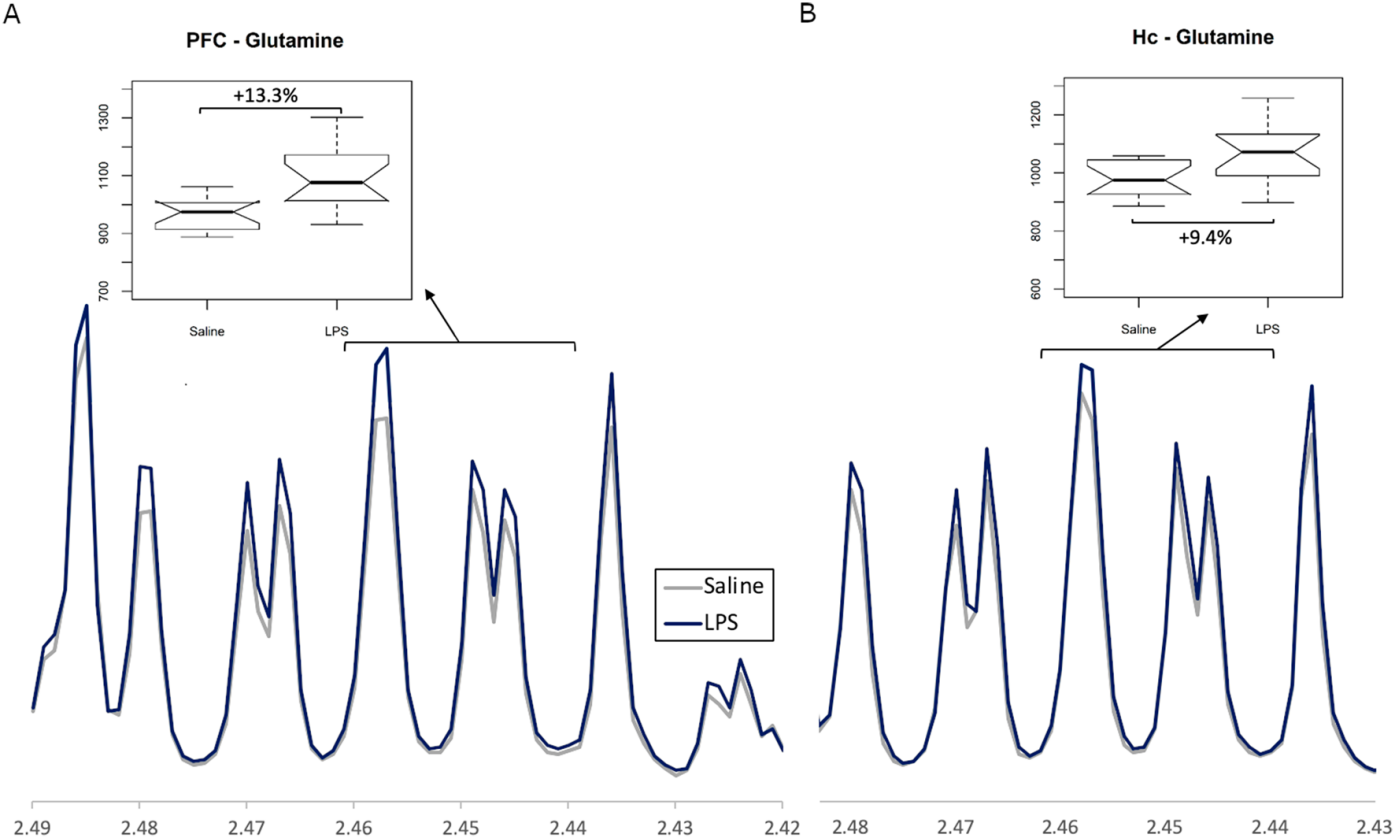
Representative images of NMR spectra showing group differences between a glutamine bin in (**A**) prefrontal cortex tissue (PFC) and (**B**) Hippocampal tissue (Hc). Spectra expressed as average of treatment group.

The key components contributing to the models were identified based on their VIP scores and summarized in Table 2. In both brain regions, LPS treatment resulted in significant increases in glutamine levels (Fig. 7A and B) and in N-acetyl aspartate (NAA) levels. Similar to the inflammatory cytokine levels, more pronounced changes were observed in the prefrontal cortex compared to the hippocampus. In the prefrontal cortex, LPS treatment also resulted in a significant decrease in alanine, glutamate, serine and histidine/phenylalanine, and was accompanied by a significant increase in glycerol levels.

**Table 2:** Summary of the key aqueous-phase metabolites driving the discrimination between LPS and saline treatment groups, and their direction of change compared to saline.

Key brain metabolites	Hippocampus (LPS vs Saline)	Frontal cortex (LPS vs saline)
Alanine		↓**
Glutamate		↓*
Glutamine	↑**	↑***
N-acetylaspartate	↑**	↑***
Glycerol		↑**
Serine		↓***
Histidine/Phenylalanine		↓**

*p < 0.05; **p < 0.01; ***p < 0.001.

Validation of metabolomics methods through spectral relative standard deviation values^34^, and mouse weights per treatment group can be found in supplementary information (Figs. S2 and S3).

## Discussion

The current study has demonstrated that behavioural despair in mice observed 24 h after an injection of LPS, was associated with reduced glutamate and increased glutamine in the prefrontal cortex, and a decrease in lipids and glucose in the plasma. The latter changes may have been a consequence of elevated circulating isoleucine (see below for discussion of this point). In addition, an observed increase in plasma creatine provides further evidence of persistent altered metabolism following inflammation.

### LPS Inflammation model and metabolomics

Results from the gene expression and behavioural analyses are in line with previous reports of the consequences of LPS-induced neuroinflammation^16,32,33,38,62,63^. Here, depressive-like behaviour, indicated by immobility time in the FST (Fig. 2A), and elevated levels of pro-inflammatory cytokines in the brain (Fig. 3C and D) were found 24-h post-LPS administration and after evidence for the presence of peripheral inflammation had subsided (Fig. 3B). While distance travelled was significantly lower in the LPS group compared to saline controls (Fig. 2C), time spent in the centre zone was also significantly lowered in the LPS group (Fig. 2D). Therefore, it is possible that LPS-treated mice displayed greater anxiety-like behaviour and travelled less in response to the novel environment^46^. We are confident that our model replicates previous findings, and that metabolomics changes observed are relevant to other behavioural outcomes observed in other studies using similar models.

Using this model, we have also shown that the metabolite profile is significantly perturbed in plasma, hippocampus, and prefrontal cortex, which suggests that the plasma metabolite changes are downstream of the acute inflammatory response to LPS and persist post-inflammation. This also suggests that there may be a link between the sustained expression of the classical APPs, such as SAA-2, in the periphery after the proinflammatory cytokines have returned to normal, and the longer-lived CNS metabolite changes^64^. The extended expression of cytokines in the brain may also influence the concentration of brain metabolites we are able to detect with NMR.

Analysis of gene expression in the hippocampus and prefrontal cortex suggests that the prefrontal cortex appears more susceptible to LPS-induced neuroinflammation, as both the mRNA levels of TNFα and IL-1b were significantly increased. This is consistent with our previous work^65,66^. A greater number of altered metabolites were also detected through NMR spectroscopy in the prefrontal cortex, compared to the hippocampus.

### Brain glutamine and glutamate

Glutamate is the main excitatory neurotransmitter in the brain. Its availability is tightly controlled by the glutamate-glutamine shuttle, where glutamate is synthesized from glutamine by glutaminase, while glutamine synthetase metabolizes glutamate to glutamine in astrocytes^67^. Dysregulation of glutamatergic signalling has been linked to some psychiatric disorders^68^, and a recent meta-analysis identified cortical glutamate to be reduced in the PFC^69^. Increasingly, the glutamate network in the brain is being viewed as both a primary mediator of neuropsychiatric pathology and therapeutic target for treating depressive disorders^70–72^. Hashimoto et al. have shown that the ratio of glutamine to glutamate in CSF is increased in patients with major depression, in a manner that correlates with the severity of depressive symptoms^73^. Other studies have reported reduced glutamate levels in the anterior cingulate cortex^74^ and PFC^75^ in humans with major depression. In our model of LPS-induced depressive-like behaviour, we found increased levels of glutamine in the two brain regions studied, and reduced glutamate in the prefrontal cortex. Together, these observations may suggest that an optimal equilibrium between central glutamate and glutamine metabolism exists, and that dysfunctional glutaminergic signalling rather than total brain serotonin levels may be the link between human major depressive disorder and LPS-induced depressive-like behaviours in mice^76^. Further studies are required to investigate the precise mechanism behind the observed reduction of glutamate:glutamine ratios, and to determine whether a causal link exists between the sustained increase in brain pro-inflammatory cytokine expression and altered glutamate and glutamine levels. It would also be interesting to see how these metabolite levels respond to conventional and novel antidepressant treatments.

### Other amino acids linked to glutamate neurotransmission

The administration of LPS reduced the level of DL-serine in the prefrontal cortex, an amino acid which, as the L-enantiomer, is involved in ceramide synthesis^77^, and as D-serine, is an essential co-agonist of brain NMDAR subunits^78^. Reduced serine levels following LPS may suggest that NMDARs are likely to be impaired. However, we and others have demonstrated elevated NMDAR subunits following LPS which suggests increased receptor function^37,47^. Therefore, a reduction in serine might be a compensatory mechanism to reduce NMDAR activity. Most likely, decreased serine levels together with the decrease in the glutamate/glutamine ratio is likely to have resulted in compensatory changes in NMDAR expression and function.

NAA has been associated with lipid synthesis and myelination by oligodendrocytes, but may also function as a reservoir for neurotransmitter synthesis and energy production^79–81^. The increase of central NAA after LPS administration is interesting and has recently been associated with depression in humans^82^. While depressive symptoms in humans are more commonly associated with reduced NAA levels^83^, increased NAA here may also be a remnant of the increased central energy conservation and storage in the brain following sickness behaviour induced by LPS^84^. The increase in glycerol concentration, which is known to be an important oxidizable substrate in the brain^85^, also suggests that there is a decrease in the utilisation of energy sources rather than a decrease in supply owing to the altered concentration of circulating glucose.

Phenylalanine is also reduced in inflammation-associated depression^86,87^. This is consistent with the current finding that LPS reduced this aromatic amino acid in the prefrontal cortex. Moreover, phenylalanine competitively inhibits NMDAR activity^88,89^, and so its central reduction might be expected to enhance NMDAR-mediated glutamate signalling and thus impair normal emotional behaviour. This notion is in-keeping with the aforementioned LPS-mediated increase in NMDAR activity^37^. It is also important to note that phenylalanine is metabolised to tyrosine, a precursor of dopamine. Acute phenylalanine depletion can impair reward-processing linked to central dopamine function^90^, though changes in tyrosine were not observed in the current study. Our metabolomic data, therefore, implicate changes in neuroactive amino acids as mediators of inflammation-induced depressive-like behaviour in mice.

### Plasma lipoproteins

A strong association exists between inflammation, the APR and lipoproteins. The APR is induced at the onset of inflammation^64^, and causes metabolic changes that result from the breakdown of serum proteins and synthesis of APP. One of the major APPs synthesized is acute-phase SAA, an apolipoprotein involved in immune cell recruitment and localisation, and the induction of pro-inflammatory cytokine expression^91–93^. The current study has shown that a dramatic increase in peripheral acute-phase SAA expressed in the liver after LPS administration (Fig. 3A) was paralleled by a decrease in plasma lipoprotein resonances (Fig. 5A), which is consistent with acute-phase SAA-mediated reductions in high density lipoprotein (HDL) levels observed by others^94^. Another APP upregulated by inflammation is endothelial lipase, which facilitates phospholipid hydrolysis^95^, and further supports the notion of inflammation and APR-mediated reduction in plasma lipoprotein levels observed. We substantiate the role of acute-phase SAA in altering lipid-based systems post-inflammation, which may play a role in the effect of LPS on peripheral and central inflammation and depressive-like behaviour.

### Plasma energy metabolism

Other key plasma metabolites (glucose, isoleucine, creatine) altered by LPS (Table 1) are involved in nutrient utilization and mitochondrial energy metabolism. Our observed decrease in glucose levels is in line with another study showing that a single administration of LPS decreased glucose utilization and production^96^. However, in the latter study, the decrease in plasma glucose levels was normalized to control levels 24 h post-injection, while the current study shows that levels remained significantly decreased after this time. It is possible that this discrepancy is due to methodological differences, as Raetzsch et al., used a handheld glucose meter. The current data suggest that the effects of LPS on glucose metabolism may last longer than originally proposed and that metabolomics analysis of plasma provides a more sensitive measure of the persistent metabolic effects of inflammation.

Both isoleucine and creatine levels increased 24 h after LPS administration. Isoleucine belongs to the family of branched chain amino acids that have been linked to many nutrient utilization functions including enhancing lipolysis and glucose consumption^97^. Thus, elevated isoleucine may underlie reduced lipid and glucose levels in the current study, and so strategies to reduce the production of this amino acid may attenuate some effects of LPS-induced inflammation. Creatine, together with phosphocreatine, are indices of skeletal muscle metabolism and are essential for adenosine triphosphate (ATP) regeneration, where phosphocreatine provides a phosphate to ADP to form ATP^98^. Rat serum ATP has been shown to be reduced 6 h after LPS, though creatine concentrations remained unaltered^99^. If ATP levels are also reduced after LPS administration in the current study, it is possible that creatine levels were elevated between 6–24 h post-LPS to restore a depleted energy reservoir. Alternatively, increased circulating creatine may be indicating continued skeletal muscle metabolism, which occurs during sepsis. Our data, consistent with previous studies, demonstrate anomalous peripheral energy metabolism, but not central energy metabolism, after LPS administration, and in addition, we show that these changes are sustained 24 h after the endotoxin injection. Whether this contributes directly to the post-inflammatory reduction in brain glutamate:glutamine ratios and exhibited depressive-like behaviours requires further exploration.

### Limitations

While ^1^H NMR metabolomics provides a vast amount of information on a range of aqueous metabolite concentrations, data on the lipid-phase metabolites are limited. In plasma samples, a spectrum of lipid species was detected only as broad peaks, and so while we were able to detect changes in overall lipoprotein concentrations, we were unable to identify the specific types of lipid affected by LPS. For the brain samples, the perchloric acid extraction protocol removes most non-polar metabolites from the sample, thus we were only able to analyse aqueous-phase, polar metabolites. Given that lipoproteins were affected by a single dose of LPS, future work will further investigate the lipoprotein composition using mass spectrometry.

Another limitation of the study was the single time-point at which LPS effects were examined. The 24 h time-point was chosen based on the established mouse inflammation model where depressive-like behaviour is observed that is not attributed to sickness behaviour, though it would be interesting to compare the metabolome at 6 h during the acute phase response to see if metabolic signatures can help describe a shift from sickness behaviour to depressive-like behaviour. While reduced time in the centre zone of the OFT and increased time floating in the FST indicated depressive-like behaviour, a longer exposure in the OFT may have provided a more accurate indication of habitual locomotor activity^100^. Other indications of anxiety-like behaviour, such as grooming behaviour, were not assessed.

This study investigated the effects of LPS on male mice, to exclude sex as a confounding factor on the effect of LPS-induced inflammation in depressive-like behaviours, which have been demonstrated to be more pronounced in male mice than females^101,102^. While the use of a male model reduced biological variation in this study, it would be interesting to investigate how LPS affects glutamine, glutamate, and other relevant metabolites in the female mouse brain to determine if this effect is sex dependent.

## Conclusions

Peripheral inflammation induced by LPS resulted in sustained changes in key plasma metabolites involved in energy metabolism, including lipoproteins, glucose, creatine, and isoleucine. In the brain, energy metabolism remained seemingly unaffected, though cortical glutamate-glutamate ratios were reduced, and amino acids potentially linked to glutamate neurotransmission (L-serine, and NAA) were altered after LPS treatment. These metabolic changes were associated with altered cytokine signalling in the brain, and depressive-like behaviour. Central glutamate-glutamine imbalances are well-documented in depressed humans and are the target of novel, rapid-acting antidepressants such as low-dose ketamine and mimetics. We propose that post-inflammatory changes after a peripheral immune challenge lead to reduced brain glutamate, which may underlie altered behaviour in this well-established LPS model of depressive-like behaviour.

### Ethics and consent to participate

This work did not involve human participants. All animal procedures were carried out in accordance with UK Home Office Animals (Scientific Procedures) Act (1986) and associated Home Office guidelines. The local Animal Welfare and Ethical Review Body at the University of Oxford approved the procedures specific to this study.

## Supplementary information

Supplementary information.

## Data Availability

The datasets used and/or analysed during the current study are available from the corresponding author on reasonable request. The authors will take responsible for maintaining availability.

## Abbreviations

LPS: Lipopolysaccharide
NMR: Nuclear Magnetic Resonance
SAM: S-adenosyl methionine
CSF: Cerebrospinal fluid
i.p.: Intraperitoneal
FST: Forced swim test
OFT: Open field test
EDTA: Ethylene Diamine Tetra Acetic Acid
NF H_2_O: Nuclease-free water
RT: Reverse transcription
qPCR: Quantitative polymerase chain reaction
SAA2: Serum amyloid A2
IL: Interleukin
TNFα: Tumour necrosis factor α
B2M: Beta-2-microglobulin
TFRC: Transferrin receptor
PCA: Principal Component Analysis
TSP: 3-Trimethylsilylpropanoic acid
1D: One-dimensional
NOESY: Nuclear Overhauser Effect Spectroscopy
CPMG: Carr-Purcell-Meiboom-Gill
RSD: Relative standard deviation
SEM: Standard error of the mean
VIP: Variable importance in projection
HMDB: Human metabolome database
COSY: Correlation spectroscopy
APP: Acute phase protein
APR: Acute phase response
NAA: N-acetyl aspartate
NMDA: N-methyl-D-aspartate
AMPA: α-Amino-3-hydroxy-5-methyl-4-isoxazolepropionic acid
HDL: High density lipoprotein
ATP: Adenosine triphosphate
