# Supplementary material for: Post-inflammatory behavioural despair in male mice is associated with reduced cortical glutamate-glutamine ratios, and circulating lipid and energy metabolites
Source: Sci Rep. 2020 Oct 8;10:16857. doi: 10.1038/s41598-020-74008-w (PMC7545201; doi:10.1038/s41598-020-74008-w)
Supplement: Supplementary file 1 — Supplementary information. [file 41598_2020_74008_MOESM1_ESM.pdf]

Supplementary Information

Post-inflammatory behavioural despair in male mice is associated with reduced cortical glutamate-glutamine ratios, and circulating lipid and energy metabolites

Shi Yu Chan[1]†, Fay Probert[2], Daniel E Radford-Smith[2], Jennifer C Hebert[1], Timothy DW Claridge[3], Daniel C Anthony[2], Philip WJ Burnet[1*]

Department of Psychiatry, University of Oxford, Oxford, OX3 7JX, UK[1]

Department of Pharmacology, University of Oxford, Oxford, OX1 3QT, UK[2]

Department of Chemistry, University of Oxford, Oxford, OX1 3TA, UK[3]

*Corresponding author: Philip WJ Burnet

Email: phil.burnet@psych.ox.ac.uk

Tel: +44 1865 618327

Address: Department of Psychiatry, Warneford Hospital, Warneford Lane, Oxford, OX3 7JX, UK

†Current Address: Psychosis Neurobiology Lab, McLean Hospital, Belmont, MA02478, USA

Supplementary Methods

Section 1: ^{1}H NMR Spectroscopy

Plasma samples: Plasma samples were defrosted on ice and centrifuged at 17,000 x*g* for 5 min at 4 °C. An equal volume of plasma was aliquoted (100 µL) into a fresh tube, and diluted to 600 µL in a 75 mM phosphate buffer (5:1 disodium phosphate Na_2HPO_4, monosodium phosphate NaH_2PO_4 in 100% D_2O, pH 7.4). The volume of plasma used was limited by the volume of plasma collected, and the volume chosen was the maximum volume available for 90% of samples. Samples with insufficient plasma volume were excluded from the analysis.

Brain tissue samples: Lyophilized samples were resuspended in 600 µL of phosphate buffer (0.2M Na_2HPO_4, 0.043 NaH_2PO_4, in 100% D_2O with 0.05 wt % 3-trimethylsilylpropanoic acid (TSP)).

Samples were then transferred to 5 mm Borosilicate Glass NMR tubes (Norrell). ^{1}H NMR spectra were acquired using a 700-MHz Bruker AVII spectrometer operating at 16.4 T equipped with a ^{1}H (13C/15N) TCI cryoprobe. Sample temperature was stable at 310 K. ^{1}H NMR spectra were acquired using a one dimensional (1D) Nuclear Overhauser Effect Spectroscopy (NOESY) pre-saturation scheme for attenuation of the water resonance with a 2 s presaturation.

An additional sequence, the spin-echo Carr-Purcell-Meiboom-Gill (CPMG) sequence, was used for plasma samples to suppress broad signals arising from large molecular weight plasma components with a τ interval of 400 µs, 80 loops, 32 data collections, an acquisition time of 1.5 s, a relaxation delay of 2 s, and a fixed receiver gain. CPMG spectra provide a measurement of small molecular weight metabolites and mobile side chains of lipoproteins in the plasma sample and were used for all further analysis of plasma samples.

Section 2: NMR Data Processing

Processing methods were adapted from published parameters (Jurynczyk et al., 2017; Probert et al., 2018). NMR spectra were imported into MestreNova (Mestrelab Research, Spain) and each spectrum was then processed manually with phase 0 (PH0) correction, baseline correction (Bernstein polynomial fit, order = 3), and referencing to an added standard (TSP referenced to δ0) for brain tissue, and an internal standard (Lactate referenced to δ1.33) for plasma. The individual spectra were then stacked, and binned (sum method, width of each integral region = 0.02ppm). Binning refers to a function where the whole spectrum is divided into bins of equal width, and all the peaks in each bin is integrated to obtain a value representing the area of all the peaks in a bin. Binned values were then exported as a spreadsheet (.xlsx) for further analysis.

Inter-individual variation was reduced by total area normalization (each bin normalized to a ratio of the individual with the lowest total area over total area of the individual) to account for any dilution error. Brain tissue samples were also further normalized to TSP (each bin normalized to a ratio of the individual with the lowest TSP area over TSP area of the individual). Finally, noise areas were removed by two methods. First, spectral relative standard deviation (RSD) values were calculated for each bin, and bins with RSD greater than 100 were removed (Parsons et al., 2009). Second, the addition of the mean and standard deviation was calculated for each bin, and the average taken for a noise region. Bins with a value lower than that of the noise region would be removed. Broadly, areas that were removed include the water peak, regions before 0.7ppm, regions after 9.38ppm, noise region 5.0 to 6.0ppm, and contamination EDTA peaks for plasma samples.

Section 3: NMR Data Analysis

Preliminary exploratory analysis: Normalized bin values were imported into SIMCA (Umetrics, Sweden). Outliers were identified with principal component analysis (PCA) scores plots with pareto scaling (van den Berg et al., 2006). Pareto scaling removes the bias given to large peaks without inflating the noise, thus allowing the detection of changes in small and medium-sized peaks. Supervised multi-variate analysis was conducted using orthogonal partial least squares discriminant analysis (OPLS-DA), which attempts to find a linear relationship between a predictor matrix (spectrometric bin values) and a response matrix (treatment groups) (Triba et al., 2015).

Model building and validation: OPLS-DA models were built in R 3.3.2 (R Core Team, 2016) using the ROPLS package (Thévenot et al., 2015) and an in-house R script using 10-fold cross validation. The total number of samples were divided into 10 groups, with nine groups used to build the model (training set) and the last group used to test the model (testing set). For each iteration of the script, each of the 10 groups was used as the testing set once, thus 10 models were built for each iteration. The 10-fold cross validation was then repeated for a total of 100 iterations, producing an ensemble of 1000 models.

The main outcome of interest is predictive accuracy of the model, reported as mean predictive accuracy of the 1000 models built with the standard error mean (standard deviation/$\sqrt{100}$ where 100 is the number of iterations). This determines how accurate the model is at predicting which group a sample in the test set belongs to, thus assessing the discriminatory power of the model.

Other parameters included the average specificity of the models, sensitivity of the models, average Q^2 values, R^2X values, and R^2Y values. The Q^2 measures the internal predictive ability of the model (the accuracy of the model on the training set) and is used to optimise the model, while the R^2 measures the goodness of fit of the model (Triba et al., 2015), with R^2X and R^2Y measuring the fraction of the variation explained by the model of the X and Y variables respectively.

To validate the OPLS-DA models, the same cross-validation process was conducted with samples randomly assigned to treatment groups. This ensemble of OPLS-DA models, representing the null distribution, was used to calculate the accuracy achieved by random chance. If the true OPLS-DA models performed significantly better than the permutation test, then the discrimination observed was unlikely to have occurred by chance and thus the results were considered significant. For significant predictive models, the variable importance in projection (VIP) scores were used to identify the key bins that were important for building the model. Bins with high VIP scores were considered to be significantly different between the treatment groups.

Metabolite identification and direction of change: Metabolites were assigned to peaks in bins with high VIP scores through a combination of literature values (Govindaraju et al., 2000; Misra & Bajpai, 2009), reference to the human metabolome database (HMDB) (Wishart et al., 2013), and confirmation with two-dimensional (2D) correlation spectroscopy (COSY). COSY provides confirmation that peaks occupying different positions on the spectrum belong to the same metabolite through cross-peaks that show a correlation between signals. The direction of

change between groups was also determined by comparing means in SPSSv20 (independent samples T-test).

Supplementary Figures

Table S1 qPCR Primer Sequences

Gene	Sequences	
TNFα	Forward	GCCTCCCTCTCATCAGTTCTAT
	Reverse	TTTGCTACGACGTGGGCTA
IL-6	Forward	CTGCAAGAGACTTCCATCCAGTT
	Reverse	GAAGTAGGGAAGGCCGTGG
IL-1β	Forward	CAACCAACAAGTGATATTCTCCATG
	Reverse	GATCCACACTCTCCAGCTGCA
SAA2	Forward	GCCATGGAGGGTTTTTTCATT
	Reverse	CCTTTGGGCAGCATCATAGTTC
IL-10	Forward	TGGACAACATACTGCTAACC
	Reverse	GGATCATTTCCGATAAGGCT
TFRC	Forward	TGGGTCTAAGTCTACAATGGCT
	Reverse	CCCTCATGACGAATCTGTTTG
B2M	Forward	CATGGCTCGCTCGGTGACC
	Reverse	AATGTGAGGCGGGTGGAACTG

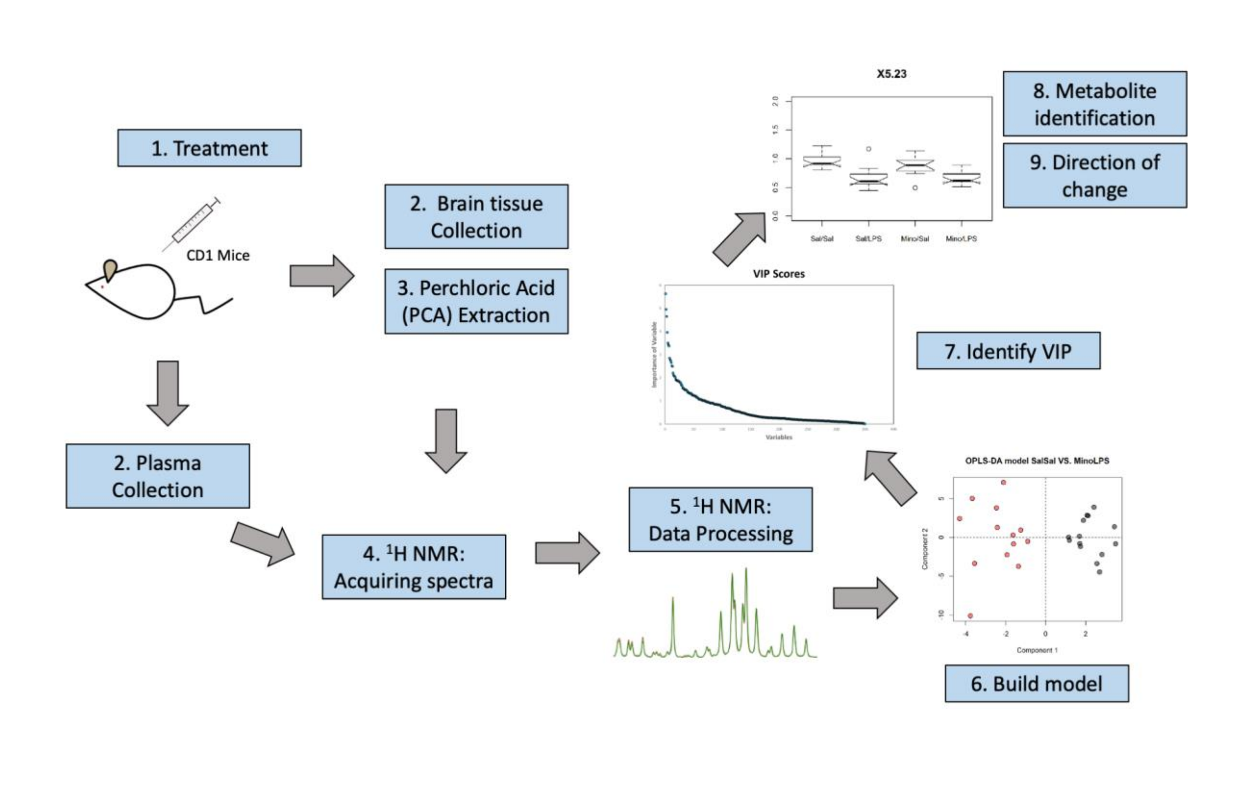

Fig S1 Metabolomics methodology: Summary of sequence of events. Original artwork was provided by Dr Biyan Zhang, A*STAR, Singapore.

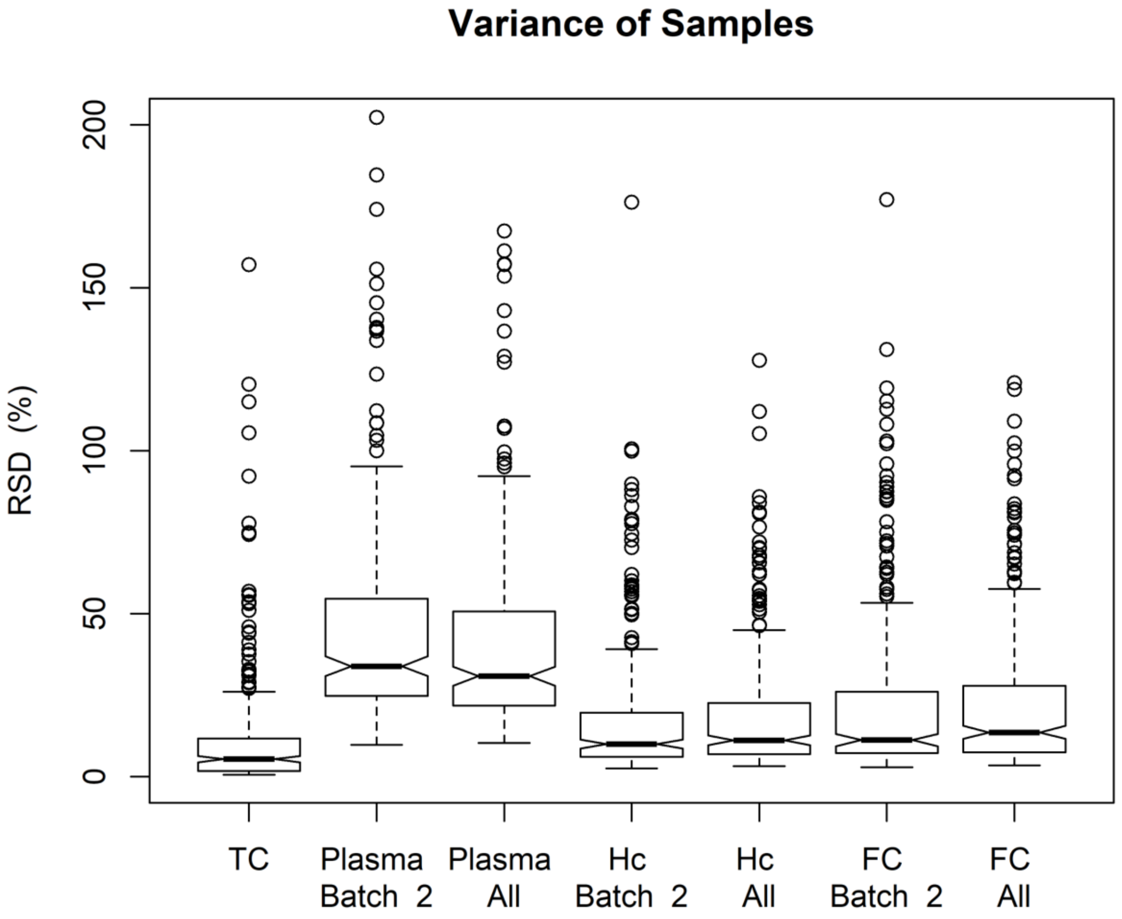

	Technical Controls	Plasma		Hippocampus		Frontal Cortex	
		Batch 2	All	Batch 2	All	Batch 2	All
Min	0.5582	9.689	10.26	2.546	3.196	2.818	3.443
1st Quartile	1.7425	24.724	21.81	6.108	6.906	7.159	7.507
Median	5.3414	33.834	30.79	9.959	11.095	11.178	13.525
Mean	12.355	93.159	55.33	18.588	19.404	23.093	22.928
3rd Quartile	11.6467	54.369	50.7	19.544	22.485	25.99	27.632
Max	157.1159	9555.778	1078.09	176.188	127.673	177.045	120.853

Fig S2 (A) Boxplot (limited to 200% RSD) and (B) Summary of spectrum wide RSD values of different cohorts.

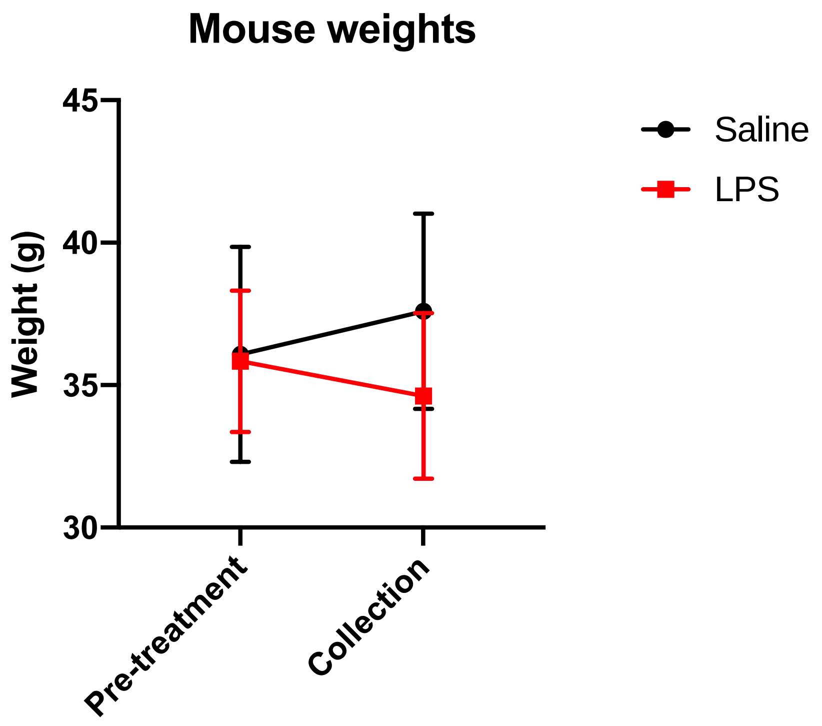

Fig S3 Weight change of mice (g) before treatment and on the day of sample collection (24 hours after LPS administration) displayed as mean per treatment group with error bars displaying standard deviation. There was no interaction effect between treatment and time ($F_{1,35} = 1.80$, $p = 0.189$), indicating that mouse weights were not significantly different between treatment groups 24 hours after LPS administration.

Plasma – Saline/LPS

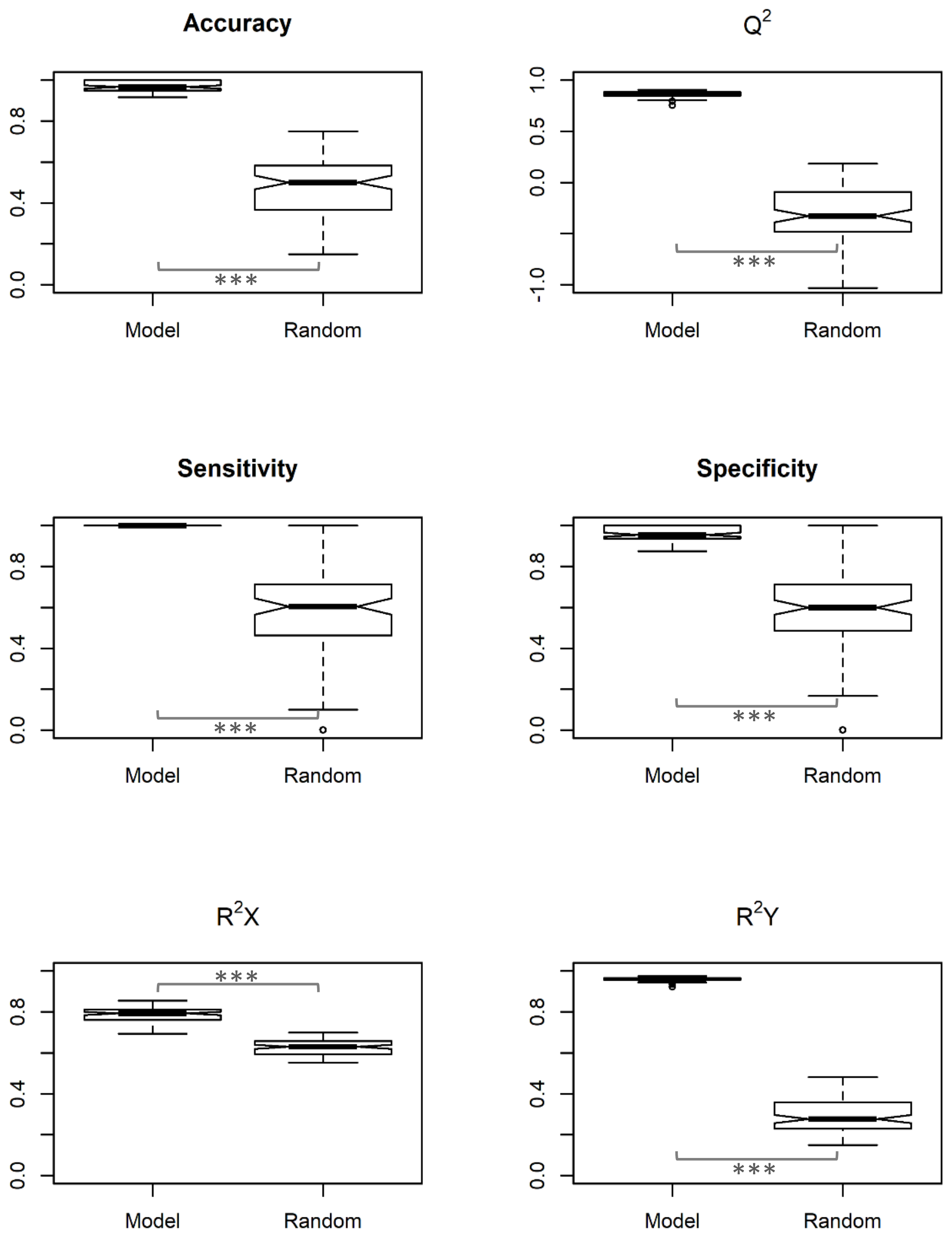

Fig S4 Comparisons between the plasma Saline/LPS OPLS-DA model (Model) and a randomly permuted model (Random). On all counts (Accuracy, Q^2, Sensitivity, Specificity, R^2X, and R^2Y), the OPLS-DA model was significantly better.

Prefrontal Cortex – Saline/LPS

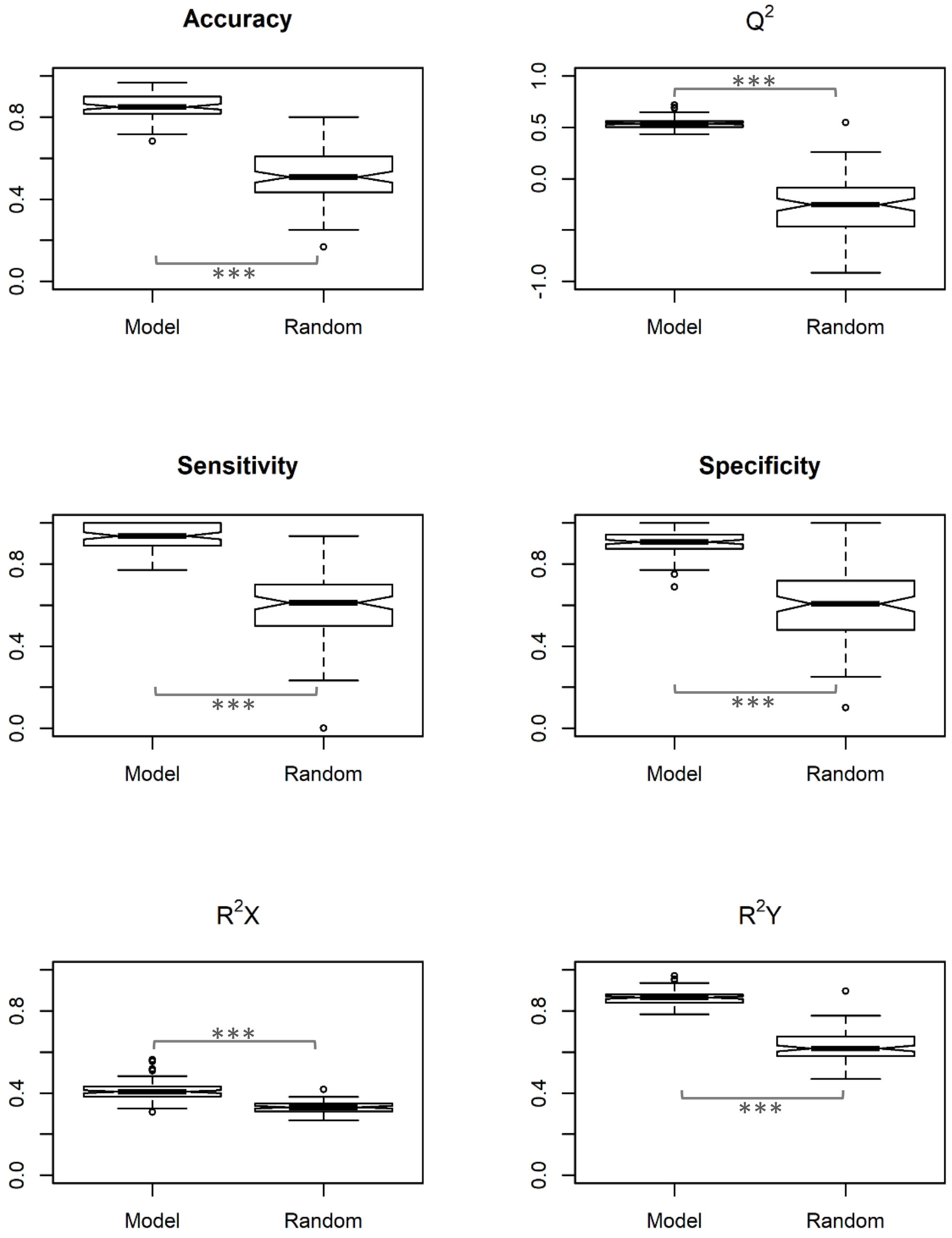

Fig S5 Comparisons between the prefrontal cortex Saline/LPS OPLS-DA model (Model) and a randomly permuted model (Random). On all counts (Accuracy, Q^2, Sensitivity, Specificity, R^2X, and R^2Y), the OPLS-DA model was significantly better.

Hippocampus – Saline/LPS

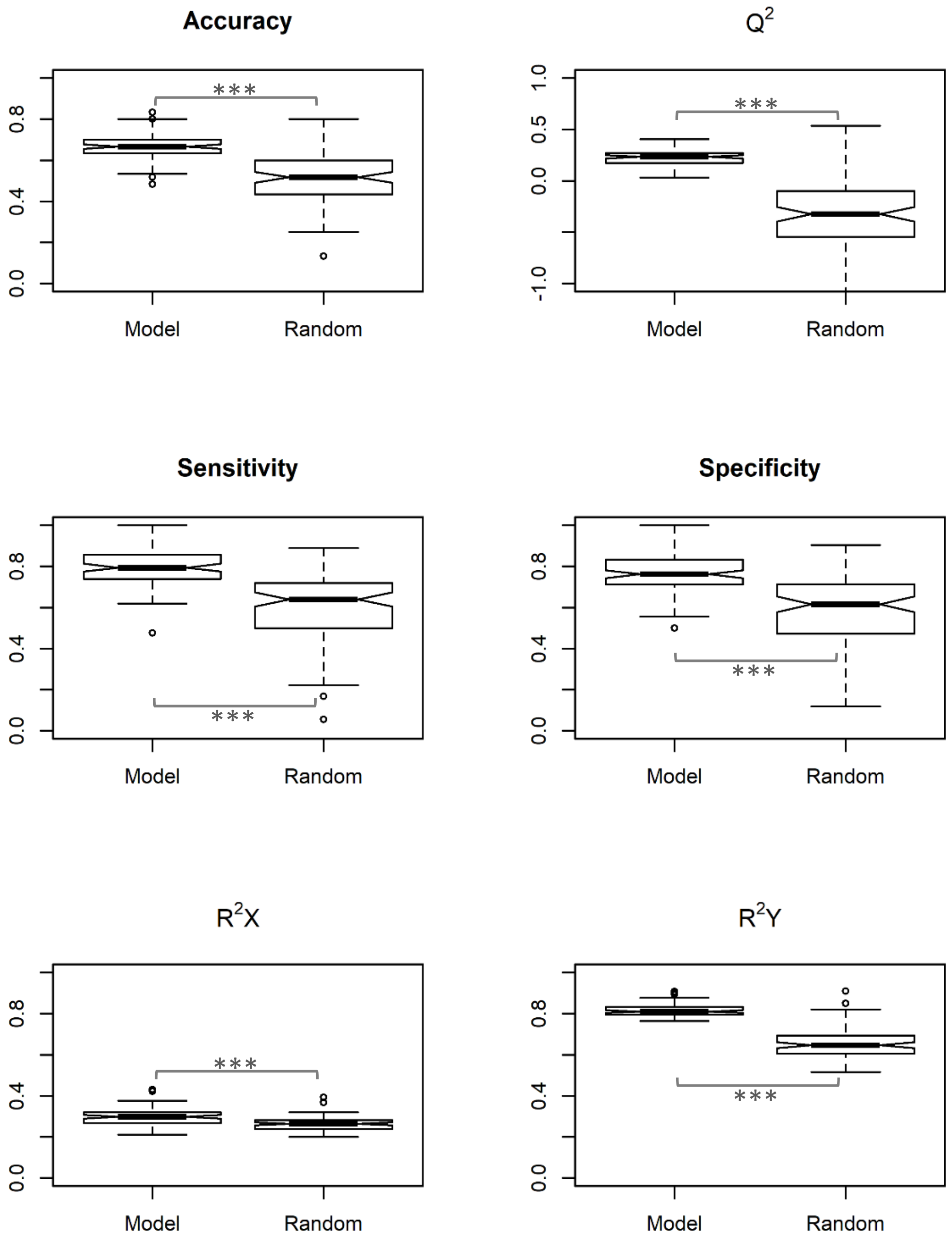

Fig S6 Comparisons between the hippocampus Saline/LPS OPLS-DA model (Model) and a randomly permuted model (Random). On all counts (Accuracy, Q^2, Sensitivity, Specificity, R^2X, and R^2Y), the OPLS-DA model was significantly better.

Table S2: Summary of Saline/LPS OPLS-DA model and randomly permuted model measures

Saline/LPS		Accuracy		Sensitivity		Specificity		Q2		R2Y		R2X	
		Model	Rand	Model	Rand	Model	Rand	Model	Rand	Model	Rand	Model	Rand
Plasma	Summary	97%	48%	100%	59%	97%	60%	0.86	-0.31	0.960	0.293	0.787	0.627
	T test	<0.001 ***		<0.001		<0.001		<0.001		<0.001		<0.001	
	K.S. Test	<0.001		<0.001		<0.001		<0.001		<0.001		<0.001	
Hc	Summary	67.1%	50.7%	78.9%	60.2%	76.5%	59.6%	0.225	-0.343	0.816	0.653	0.297	0.262
	T test	<0.001 ***		<0.001		<0.001		<0.001		<0.001		<0.001	
	K.S. Test	<0.001		<0.001		<0.001		<0.001		<0.001		<0.001	
PFC	Summary	85.3%	51.1%	92.9%	59.9%	90.2%	60.4%	0.537	-0.315	0.864	0.622	0.411	0.330
	T test	<0.001 ***		<0.001		<0.001		<0.001		<0.001		<0.001	
	K.S. Test	<0.001		<0.001		<0.001		<0.001		<0.001		<0.001	
